# For which infants with viral bronchiolitis could it be deemed appropriate to use albuterol, at least on a therapeutic trial basis?

**DOI:** 10.15586/aei.v49i1.12

**Published:** 2021-01-02

**Authors:** Carlos E. Rodríguez-Martínez, Gustavo Nino, Jose A. Castro-Rodriguez, Ranniery Acuña-Cordero, Monica P. Sossa-Briceño, Fabio Midulla

**Affiliations:** a Department of Pediatrics, School of Medicine, Universidad Nacional de Colombia, Bogota, Colombia; b Department of Pediatric Pulmonology and Pediatric Critical Care Medicine, School of Medicine, Universidad El Bosque, Bogota, Colombia; c Division of Pediatric Pulmonary, Sleep Medicine and Integrative Systems Biology, Center for Genetic Research, Children’s National Medical Center, George Washington University, Washington, DC, USA; d Department of Pediatric Pulmonology, School of Medicine, Pontificia Universidad Catolica de Chile, Santiago, Chile; e Department of Pediatric Pulmonology, Hospital Militar Central, Department of Pediatrics, School of Medicine, Universidad Militar Nueva Granada, Bogota, Colombia; f Department of Internal Medicine, School of Medicine, Universidad Nacional de Colombia, Bogota, Colombia; g Department of Pediatrics, Sapienza University, Rome, Italy

**Keywords:** bronchiolitis, viral bronchiolitis phenotyping, clinical practice guidelines, phenotype-specific treatment

## Abstract

Although there is increasing evidence showing that infants with viral bronchiolitis exhibit a high degree of heterogeneity, a core uncertainty shared by many clinicians is with regard to understanding which patients are most likely to benefit from bronchodilators such as albuterol. Based on our review, we concluded that older infants with rhinovirus (RV) bronchiolitis, especially those with a nasopharyngeal microbiome dominated by *Haemophilus influenzae*; those affected during nonpeak months or during non-respiratory syncytial virus (RSV) predominant months; those with wheezing at presentation; those with clinical characteristics such as atopic dermatitis or a family history of asthma in a first-degree relative; and those infants infected with RSV genotypes ON1 and BA, have the greatest likelihood of benefiting from albuterol. Presently, this patient profile could serve as the basis for rational albuterol administration in patients with viral bronchiolitis, at least on a therapeutic trial basis, and it could also be the starting point for future targeted randomized clinical trials (RCTs) on the use of albuterol among a subset of infants with bronchiolitis.

## The rationale for phenotype-specific treatment of viral bronchiolitis

Viral bronchiolitis is the most important cause of lower respiratory tract infection in children during the first year of life and the leading cause of hospitalization among infants younger than 6 months.^1^ In addition to the significant clinical burden of viral bronchiolitis on patients,^2^ the disease is usually associated with substantial direct and indirect costs, not only for healthcare systems but also for families and society as a whole.^3^

For decades, it has been a well-established position that bronchiolitis treatment is mostly supportive, focusing only on observation, hydration, and oxygen supplementation. Although prior evidence-based medicine (EBM) clinical practice guidelines (CPGs), such as the 2006 American Academy of Pediatrics (AAP) bronchiolitis CPG, recommended the use of bronchodilators on a trial basis,^4^ the latest EBM-based CPGs on viral bronchiolitis no longer recommend a trial of bronchodilators.^5,6^ The most common arguments put forth for this new recommendation are the greater strength of the evidence demonstrating no benefit in the bronchiolitis population as a whole and that there is no well-established way to determine an “objective method of response”.^5^ However, there is increasing evidence showing that the implicit assumption that all affected infants with “viral bronchiolitis” are a homogeneous group of patients with the same clinical condition is not valid. Data from several studies have shown that affected patients exhibit a high degree of heterogeneity in their clinical presentation, immune responses, and molecular immune signatures, and show a high probability of distinct responses to different therapeutic options (phenotype-specific treatment strategies).^7^

However, despite this novel and promising way of understanding the disease, a core uncertainty shared by many clinicians is with regard to understanding which patients are most likely to benefit in some way from each of the currently available medications (i.e., bronchodilators such as albuterol).^8^ Just as it is inappropriate to use albuterol indiscriminately for all patients with the diagnosis of “viral bronchiolitis”, it would also be inappropriate to fail to administer it to patients who could benefit from it. Using albuterol based on specific patient characteristics or biomarkers, that is, the use of personalized precision medicine, instead of a “one-size-fits-all” treatment strategy could help contribute to the treatment of “viral bronchiolitis” in a more cost-effective way. Although the most appropriate way to reach the above-mentioned goal is to conduct randomized clinical trials (RCTs) using biomarkers to stratify patients most likely to respond to treatment with albuterol, on a provisional basis it could be of central importance to identify the patient profile that has the greatest likelihood of benefit from albuterol based on current knowledge of immune responses and molecular immune signatures.

This patient profile could serve as a basis for rational albuterol administration in patients with “viral bronchiolitis”, at least on a therapeutic trial basis.^9^ This is because despite the lack of RCTs demonstrating a beneficial effect of albuterol in certain phenotypes of viral bronchiolitis (i.e., phenotypes with pro-asthmatic or Th2 immune responses) up to now, there is a scientific rationale that supports this practice. Seumois et al.^10^ examined the correlation between expression of asthma-specific genes and lung physiological measures such as forced expiratory volume in 1s (FEV1), bronchodilator reversibility (BDR) following albuterol treatment, and methacholine challenge, and found a moderately good correlation (Spearman correlation coefficient ranging from 0.351 to 0.40, p<0.05) between BDR and transcriptional profiling of Th2 cells (ZBTB10, SGK1, and GABARAPL1), suggesting that the molecular program in circulating Th2 cells may influence BDR. We, therefore, aimed to summarize and analyze the current evidence that could help identify possible phenotypes or subgroups of bronchodilator responders among infants with “viral bronchiolitis”.

## Causative viral agent

Respiratory syncytial virus (RSV) and rhinovirus (RV) are the most important causative agents of viral bronchiolitis.^11^ Notably, Human rhinovirus (HRV)-infected infants display a different acquired immunological response compared to infants with RSV bronchiolitis, with a predominant Th-2 polarization, exhibiting significantly higher Th2 cell frequencies, significantly higher Th2 index (calculated as the Th2/Th1 ratio),^12^ and increased airway interferon lambda receptor 1 (IFNL1R) transcript levels.^13^ Additionally, in infants with viral bronchiolitis, HRV and RSV infections have different nasal airway microRNA profiles associated with a nuclear factor kappa-light-chain-enhancer of activated B cell (NFκB) signaling. Hasegawa et al.^14^ demonstrated that infants with HRV have higher levels of NFκB induced type-2 cytokines (IL-10 and IL-13) compared to those with RSV infection. Along the same lines, there is evidence showing that RSV and RV bronchiolitis are associated with significantly different nasopharyngeal metabolomes and bacterial metagenomes. Stewart et al.^15^ demonstrated that RSV and RV are associated with different metabolic pathways and that the associated bacterial functional capacity is derived primarily from *Streptococcus pneumoniae* in RSV bronchiolitis and from *Haemophilus influenzae* in RV bronchiolitis. This evidence supports the concept that viral bronchiolitis should be considered to be a heterogeneous disease that involves a complex interplay among virus, microbiome, and host. Supporting this concept, Zhang et al.^16^ demonstrated that in infants <6 months of age during the first episode of severe RSV bronchiolitis, the relative abundance of *Haemophilus*, *Moraxella*, and *Klebsiella* was higher in infants who later developed recurrent wheezing by the age of 3 years than in those who did not. Also, in comparison to infants with RSV bronchiolitis, those infected with RV are more likely to be older, have a prior history of eczema, be treated with systemic corticosteroids,^17^ have a significantly shorter length of stay,^18^ and have an increased risk of subsequent development of childhood asthma.^19,20^ Additionally, it has been estimated that there is a 25% population-based increased risk of early childhood asthma following infant bronchiolitis occurring during RV-predominant months compared to asthma following infant bronchiolitis during RSV-predominant months.^21^ Interestingly, new concepts have recently emerged concerning a possible significant role of HRV in asthma inception. Recent genome-wide association studies (GWAS) have shown that the ORMDL3 locus in chromosome 17q, the most highly replicated GAWS finding for asthma to date, seems to exert its effects by increasing susceptibility to RV in early life.^22^ The hypothesis emerging from these data is that infants with RV-bronchiolitis, especially those with a nasopharyngeal microbiome dominated by *H. influenzae*, could be good candidates for administering or testing albuterol in an attempt to improve clinically important outcomes.

## The epidemic-prone season

Cangiano et al.,^23^ after dividing infants according to hospitalization during the peak months and nonpeak months of the year (with RSV infections predominating during the peak months), found significant differences in terms of risk factors for respiratory diseases: infants hospitalized during the peak months had a lower family history of asthma, had more smoking mothers during pregnancy, had a slightly higher percentage of being breastfed, had a lower number of blood eosinophils, and had higher clinical severity scores. The authors hypothesized that infants hospitalized during the peak months of bronchiolitis epidemics and those hospitalized in nonpeak months might reflect two different populations of infants. The same group performed additional analyses aimed at testing the hypothesis that the balance of type-1/type-2 immune responses differs between these two populations of infants. They found that infants hospitalized during the nonpeak months had a significantly higher percentage of CD4 T cells producing IL-4, a slightly lower percentage of CD8 T cells producing IFNγ, and a significantly higher Th2 polarization than infants hospitalized during the peak months.

The authors concluded that there are at least two different bronchiolitis phenotypes: previously healthy full-term infants, hospitalized with RSV bronchiolitis during the peak months, and infants with a possible genetic predisposition to atopy, hospitalized during the nonpeak months.^24^ Although these findings could be explained by the predominance of RV infections that may occur during the non-RSV-predominant months, this seems not to be the case always, because, although in the 10 consecutive annual epidemics evaluated RSV was detected mostly during peak months, RV was equally distributed during the seasons.^23,24^ Although replication is needed, all these evidences collectively support the idea that albuterol could be used or tested in infants suffering from viral bronchiolitis during nonpeak months or during non-RSV-predominant months.

## Age of patients

Dumas et al.^25^ analyzed data from two prospective, multi-center cohorts of children younger than 2 years old hospitalized with viral bronchiolitis. Severe bronchiolitis profiles were determined by latent class analysis, classifying children based on clinical factors and viral etiology. Among the four clinical profiles (phenotypes) identified, it is worth highlighting “Profile A”: patients characterized by a history of wheezing and eczema, wheezing at the emergency department (ED) presentation, and RV infection.

Children in this profile were also more often boys, more often older (>6 months), and more often had a parental history of asthma. These results support the hypothesis that children in this profile present early signs of asthma and could be potential responders to albuterol. Additionally, older meta-analyses of the efficacy of bronchodilator therapy in viral bronchiolitis, which showed modest short-term improvements in some clinical features included older infants (greater than 1 year of age).^26,27^ Interestingly, two independent studies aimed at a better understanding of predictors of prescription of albuterol among clinicians who attend infants with viral bronchiolitis have identified the age of patients as an independent predictor of prescription of albuterol, with the older patients being more likely to be prescribed this bronchodilator therapy.^28,29^

Altogether, these results support the concept that the older the patient with viral bronchiolitis, the higher the probability of obtaining clinical benefit with albuterol use.

## Bedside clinical parameters

Although infants requiring the first hospitalization due to viral bronchiolitis have a high risk of developing subsequent recurrent asthma-like symptoms, few studies have evaluated the utility of bedside clinical assessment (e.g., wheezing, subcostal retractions, or hypoxemia) for predicting the risk of recurrence. Our group evaluated both individual risk factors and bedside clinical parameters for predicting recurrence after viral lower respiratory tract infection (LRTI) hospitalization in a cohort of young children. Wheezing and family history of asthma were identified as the only two independent predictors of recurrence after viral LTRI hospitalization in young children.^30^ Our group went further, evaluating nasal airway levels of type-2 cytokines (IL-13/IL-4). Children wheezing at first hospitalization and with at least one subsequent episode of asthma-like symptoms had higher nasal airway levels of type-2 cytokines (IL-13/IL-4), with no significant differences in other cytokines.^31^ Collectively, these data indicate that infants with wheezing at presentation have a higher probability for developing subsequent recurrent asthma-like symptoms with higher nasal airway levels of type-2 cytokines; therefore, suggesting a potential role of bronchodilators such as albuterol in treating their condition.

## Clinical characteristics

Despite the recommendations against the use of bronchodilators given in the more recent CPGs on viral bronchiolitis,^5,6^ there is increasing evidence showing that these recommendations have not yet had a major impact on all physicians’ behavior, not necessarily due to a lack of awareness of these guidelines.^32^ Specifically, there are reports showing rates of use of bronchodilators ranging between 18 and 90%, with substantial differences between countries and even between hospitals in the same country.^29,33,34^

Although clinicians may have decided to use albuterol and other bronchodilators intuitively, or perhaps even instinctively in patients with certain clinical characteristics, there is some evidence supporting this therapeutic decision. Specifically, Alansari et al.^35^ reported that dexamethasone with salbutamol shortened the time for readiness for infirmary discharge during bronchiolitis episodes in patients with atopic dermatitis or a family history of asthma in a first-degree relative. Additionally, atopic dermatitis was identified as an independent predictor of inappropriate use of diagnostic tests and management of bronchiolitis, which in turn was mainly due to the use of inhaled or nebulized Beta-2 agonists.^34^ Interestingly, supporting this practice, there is evidence showing that atopic dermatitis aggravates allergic airways inflammation in patients with acute viral bronchiolitis.^36^

All this evidence collectively suggests that infants suffering from viral bronchiolitis with clinical characteristics such as atopic dermatitis or a family history of asthma in a first-degree relative could benefit from therapy with albuterol.

## Respiratory syncytial virus genotype

Midulla et al.^37^ studied the RSV genotype distribution, clinical presentation, and disease severity in 998 previously healthy term infants less than 1-year-old hospitalized for bronchiolitis over 12 epidemic seasons. Stratifying data according to genotypes NA1, ON1, and BA showed that when compared to infants infected with genotype NA1, those infected with genotype BA had less severe symptoms and more frequently had eosinophilia and familiar history of asthma. Similarly, although to a lesser extent, those with the ON1 genotype had more risk factors for asthma and atopy than infants infected with genotype NA1. Although future replication studies are required, these findings, therefore, imply that the less virulent RSV genotypes (ON1 and BA) preferentially cause bronchiolitis in infants with a possible genetic predisposition toward asthma and atopy. Accordingly, even though genotypification of RSV is not readily available for clinical use at this time, these findings suggest a potential role of bronchodilators such as albuterol in treating infants infected with RSV genotypes ON1 and BA.

## Concluding remarks

Despite the recommendation against the use of albuterol in infants with viral bronchiolitis given in the main CPGs on viral bronchiolitis, there is increasing evidence showing that this recommendation is not being followed by a not inconsiderable percentage of pediatric care providers. Although at first glance this use of albuterol might appear inappropriate, there is increasing evidence showing that infants with viral bronchiolitis exhibit a high degree of heterogeneity in their clinical presentation, immune responses, and molecular immune signatures, as well as probably distinct responses to different therapeutic options such as albuterol. Identifying the patient profile that could benefit most from treatment with albuterol is essential for rational albuterol use in patients with viral bronchiolitis, at least on a therapeutic trial basis.

Based on our review, we concluded that older infants with RV-bronchiolitis, especially those with a nasopharyngeal microbiome dominated by *H. influenzae*; those affected during nonpeak months or non-RSV-predominant months; those with wheezing at presentation; those with clinical characteristics such as atopic dermatitis or a family history of asthma in a first-degree relative; and those infants infected with RSV genotypes ON1 and BA have the greatest likelihood of benefit from albuterol (Table 1).

Presently, this patient profile could serve as a basis for rational albuterol administration in patients with viral bronchiolitis, at least on a therapeutic trial basis, and it could also be the starting point for future targeted RCTs on the use of albuterol among a subset of infants with bronchiolitis.

## Figures and Tables

**Table 1:** Proposal for albuterol administration in patients with viral bronchiolitis, at least on a therapeutic trial basis.

	Pros for use of albuterol	Cons for use of albuterol
Viral agent	RV^12,14,19–21^	RSV
RSV genotype	RSV genotype ON1 and BA^37^	RSV genotype NA1
Seasonal period	Nonpeak months or non-RSV-predominant months^23,34^	Peak months for RSV
Nasal microbiome	*Hemophilus influenzae* ^15,16^	*Streptococcus pneumoniae*
Clinical presentation	Older age (>6–12 months)^25–29^	Younger
	Wheezing^30,31^	Crackles
	Atopic dermatitis^34,35^	-
	History of asthma in first-degree family^30,35^	-

RV: rhinovirus; IL: interleukine; IFNγ: interferon gamma; IFNL 1R: interferon lambda receptor 1; NFκB: nuclear factor kappa-light-chain-enhancer of activated B cell; RSV: respiratory syncytial virus.
